# Case report: An unusual case of multisite embolism in a patient with adenovirus pneumoniae

**DOI:** 10.3389/fmed.2022.939102

**Published:** 2022-09-06

**Authors:** Jia-Yu Mao, Hua Zhao, Na Cui

**Affiliations:** Department of Critical Care Medicine, Peking Union Medical College Hospital, Peking Union Medical College and Chinese Academy of Medical Sciences, Beijing, China

**Keywords:** adenovirus, pneumonia, left ventricular thrombus, cerebral embolism, multisite embolism

## Abstract

A 36-year-old previous healthy man presented with fever, cough, and dyspnea associated with adenovirus pneumonia. The patient developed left ventricular thrombus, pulmonary embolism and multisite embolism of undetermined etiology. Adenovirus is a rare cause of thrombotic events in immunocompetent individuals, calling for further studies for early diagnosis and management.

## Introduction

Adenovirus is a one of the most common respiratory viruses. Human adenovirus pneumonia is notorious in immunosuppressed people and may cause outbreaks of acute lung injury (1). Among military trainees, patients with Acquired Immune Deficiency Syndrome (AIDS) and transplant recipients, life-threatening adenoviral pneumonia has been documented (2–4). In addition to lung involvement, patients may develop several extrapulmonary manifestations, such as retinitis, encephalitis, hepatitis, colitis, and cystitis (5).

In 2020, Corona Virus Disease 2019 (COVID-19) stands out as the leading cause of viral coagulopathy (6). A number of thrombotic and thromboembolic complications were reported and were associated with high mortality (7–10). In the course of viral infection, a severe inflammatory response and critical illness may predispose patients to thrombotic events (11, 12). In viral infection thrombosis was shown present in various sites, such as pulmonary, proximal deep-vein, coronary and intracranial vessels (13). Thrombosis, even cardiac thrombosis in viral infection, especially adenovirus pneumonia, is extremely rare. We present an unusual case of adenovirus pneumoniae infection presenting with left ventricular thrombus, pulmonary embolism and multisite embolism.

## Case report

**FIGURE 1:**
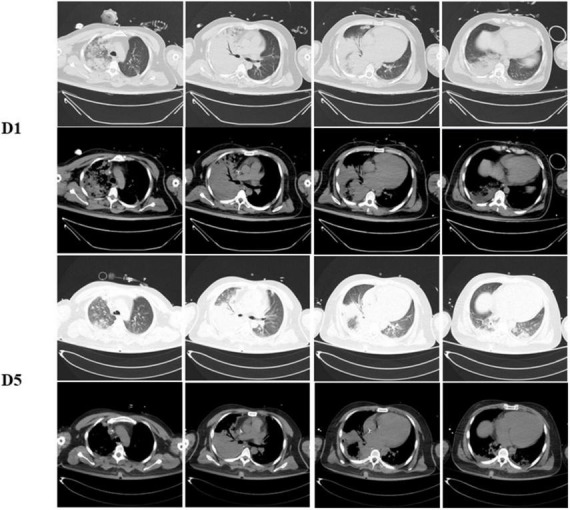
Dynamic change on computed tomography chest of the patient. Chest CT scans show with large area consolidation of the right lung on day 1 and improved after 5 days admitted.

A 36-year-old previously healthy male presented with fever, cough and expectoration for 9 days. He had no past medical history but had a history of smoking and alcohol consumption. He had a maximum temperature of 39.1°C. He revealed a blood pressure of 121/75 mmHg but with mild tachycardia and severe tachypnea of 111/min and 40/min, and oxygen saturation of 97% under 50% fraction of inspiration O_2_ (FiO_2_) supplied by a high flow nasal catheter. Chest radiograph and computed tomography (CT) scan demonstrated large area consolidation in the right lung (Figure 1). Intubation was performed, and mechanical ventilation was then administered. He was prescribed piperacillin/tazobactam, moxifloxacin and linezolid on the day of admission diagnosed as severe pneumonia.

**FIGURE 2:**
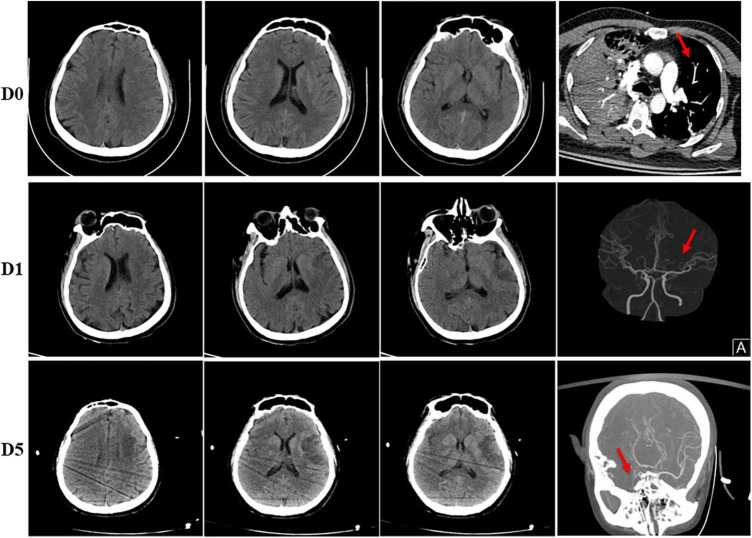
Head imaging of the patient. (D0) Normal head CT before admitted. His CTPA showed embolism of left pulmonary artery branches (red arrow). (D1) Ischemic infarction of left frontal and temporal lobes was shown in his head CT on day 1 after admitted, CTA examination showed stenosis of the left MCA (red arrow). (D5) CT scan of head on day 5 showed no obvious difference compared to day 1, however, CTA examination showed occlusion of right MCA (red arrow).

**FIGURE 3:**
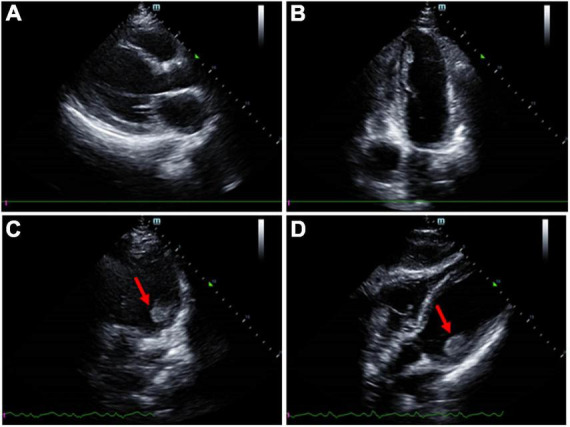
Transthoracic echocardiogram imaging of the patient. **(A)** Parasternal window parasternal long-axis view. **(B)** Apical window four-chamber view. **(C,D)** A mural thrombus was identified attached to the posteromedial papillary muscle.

After transferred to the intensive care unit, the patient exhibited onset right-sided weakness. He presented with remarkable left gaze and right-sided hemiplegia. An immediate CT scan of the head showed an acute infarction of the left frontal and temporal lobes, and computed tomography angiography (CTA) examination showed occlusion of the left middle cerebral artery (MCA) (Figure 2). D-dimer level was revealed markedly elevated at 52.26 mg/L FEU (<0.5 mg/L FEU, mg/L Forty-foot Equivalent Unit), and ultrasound of the lower extremity showed venous thrombosis of the right posterior tibial vein. His computed tomography pulmonary angiography (CTPA) showed embolism of the left pulmonary artery branches. Laboratory workup revealed slightly elevated cardiac troponin I (cTnI) at 0.29 μg/L (<0.017 μg/L) and N-terminal pro brain natriuretic peptide (NT-proBNP) at 1,461 pg/ml; his electrocardiogram (EKG) revealed non-specific manifestation. An echocardiogram revealed left ventricular (LV) thrombus with mildly reduced LV ejection fraction (36%), without concordant regional wall motion abnormality. A mural thrombus (measuring 2.8 × 1.8 cm) was identified attached to the posteromedial papillary muscle of the left ventricular apex (Figure 3).

The workup for sepsis, including testing for bacterial and fungal cultures, mycoplasma, chlamydia, legionella, tubercle bacillus, virus like cytomegalovirus, Epstein-Barr virus, and novel coronavirus was negative. The patient was confirmed diagnosing adenovirus pneumonia through metagenomics next-generation sequencing. His blood and bronchoalveolar lavage (BAL)-fluid samples were obtained, cell-free DNA was extracted and sequencing library was constructed for pipeline of bioinformatics analysis, more details could be obtained from our previous study (14). High viral loads of HAdV-B were detected in both plasma and bronchoalveolar lavage (BAL)-fluid. In addition, our sample showed high average nucleotide sequence similarity with the genome GCA_006448055.1 of Human adenovirus B strain 55, and consistency in the coverage depth profiles of the genomes of HAdV-B55.

His lymphocyte count when admitted was normal at 850/μL (800–4,000/μL) but with a mildly decreased T lymphocyte count at 696/μL (1,185–1,901/μL), among which T4# was 494/μL (561–1,137/μL) and T8# was 186/μL (404–754/μL). Other test showed elevated level of C-reactive protein (CRP) at 172.1 mg/L (<8 mg/L) and high levels of inflammatory factors, IL-6 at 42 pg/ml (<5.9 pg/ml), IL-8 at 121 pg/ml (<62 pg/ml), IL-10 at 7.4 pg/ml (<9.2 pg/ml) and TNF α at 12.7 (<8.1 pg/ml). His platelet counts and coagulation function parameters, such as activated partial thromboplastin time and prothrombin time, were in the normal range. Tests of antiphospholipid antibodies, including anticardiolipin, anti-β2-glycoprotein lupus and anticoagulant were negative. Other thrombophilia indicators, such as antithrombin III, Protein-C and Protein-S, were within the normal range. The patient’s condition gradually improved, his fraction of inspired oxygen gradually improved to 30%, and successful weaning was carried out. Consolidation of the right lung on chest radiography and CT improved 5 days after admission (Figure 1).

Therapeutic heparin was administered once diagnosing acute stroke, LV thrombus and deep venous thrombosis (DVT), with decreased D-dimer from 52.26 to 5.34 mg/L FEU. However, on the fifth day after admission, the patient experienced an acute weak pulse of the right upper limb artery, and ultrasound verified onset thrombosis of the right brachial artery. Moreover, the patient gradually developed a progressive disturbance of consciousness. His CT scan of head showed no obvious difference compared to admitted; however, CTA examination showed new onset occlusion of right MCA and right internal carotid artery, likely due to dislodgement of the left ventricular thrombus (Figure 2). A multidisciplinary consultation was involved, including representatives from Cardiology, Neurology, Vascular surgery and Intervention Clinic. Recombinant tissue plasminogen activator or thrombectomy surgery could not be administered due to his LV thrombus for an unknown cause. The patient’s family refused further treatment, and the patient was discharged from the hospital.

## Discussion

Thrombosis associated with adenovirus pneumoniae is extremely rare, especially among immunocompetent adults. Only a few cases showed extrapulmonary manifestation of adenovirus as disseminated intravascular coagulation (15) or thrombotic microangiopathy (16). We demonstrated a case of left ventricular thrombus, pulmonary embolism and multisite embolism, including cerebral embolism and artery embolism, in a patient with adenovirus pneumoniae.

The patient presented with an unusually large LV thrombus without past cardiac medical history or other positive thrombophilia indicators. The diagnosis of myocarditis could hardly be made either. A hypercoagulable state was described in the course of viral infection. Adenovirus vectors are most popular in basic vascular experiments (17). Adenoviruses seem to have the ability to directly infect the endothelium (18), and the vascular endothelium also presented specific anti-adenovirus reactions (17). Adenoviruses may also stimulate tissue factor expression to upregulate the extrinsic pathway of coagulopathy and induce procoagulant activity in infected endothelial cells (19). Direct viral endothelial injury and procoagulant activity might contribute the development of thrombosis during adenovirus infection. Moreover, proinflammatory cytokines, a condition found in COVID-19 patients, was also discussed (20). Coagulation pathways might be activated due to inflammatory cytokine release. A 17-year-old patient with COVID-19 showed improved clinical status, and the mural thrombus nearly resolved during the hospital stay after treating with tinzaparin (21). However, even when treated with anticoagulant, our patient still developed multiple site embolism repeatedly, which led to his abandonment of treatment. Because of the small number of cases, further exploration is still needed to determine the cause of the different prognoses.

Both arterial thromboembolism, including left ventricular thrombus, cerebral embolism, artery embolism, and venous thrombus, including pulmonary embolism and deep veinous thrombosis, were observed in our patient. In the critically ill patients with COVID-19, the rates of arterial and venous thromboembolism were 5 and 31% (22). A significantly high D-dimer level triggered us in an early stage to follow the clue of thrombosis and start anticoagulant therapy. D-dimer level is a sensitive indicator for identifying thrombus although it can rise in many conditions. In patients with COVID-19, D-dimer was also shown associated with the disease severity and mortality (23). The D-dimer level may also be an early warning indicator in patients with adenovirus pneumonia.

The patient in our case was previously healthy and provided no immunosuppressive background. His lymphocyte count was normal, with only a mildly decreased T lymphocyte count. Species of HAdV-55 were identified through NGS of his plasma and BALF samples. HAdV-55 was initially identified in emergent acute respiratory disease originating in China (24). Severe pneumonia has been reported in HAdV-55 compared to other serotypes, with high rates of oxygen therapy, mechanical ventilation, and mortality (25, 26). Moreover, immunocompetent adults seem to be susceptible (1). There is currently no description of thrombotic events in HAdV-55-infected patients. Further study is still needed to explore this relationship.

Some unexpected thrombotic events were developed after COVID-19 vaccinations (27). It seems that fewer thrombotic events were reported in vaccines using mRNA technology instead of adenovirus vectors (28). Direct interaction between adenovirus and blood components was hypothesized. Mechanism has been assumed recently that platelet-activating antibodies targeting the PF4–polyanion complex may account (29, 30). Overall, more research and clinical cases are still needed to clarify these findings.

Our study had several limitations. It is unfortunate that in our case the patient was discharged from the hospital after exacerbation, complete treatment and follow-up could not be performed. Second, only one patient was shown in our manuscript, more cases are still needed for further exploring occurrence rate, clinical features and prognoses of thrombotic events during adenovirus infection. Third, D-dimer level may be influenced by lots of factors, the appropriate level to identify thrombosis is still a challenge.

## Conclusion

Adenovirus is a rare cause of thrombotic events in immunocompetent individuals. This case demonstrates unusual presentations of adenovirus infection with LV thrombus, pulmonary embolism and multisite thrombotic events. We hypothesize that it may be related to viral direct endothelial injury and procoagulant activity. We aim to raise awareness that thrombotic events are not unique in COVID-19, and more attention may be drawn to unusual extrapulmonary manifestations of adenovirus infection. D-dimer levels might be an indicator in early identification and intervention concerning thrombotic events in these patients.

## Data availability statement

The original contributions presented in this study are included in the article/supplementary material, further inquiries can be directed to the corresponding author.

## Ethics statement

Written informed consent was obtained from the individual(s) for the publication of any potentially identifiable images or data included in this article.

## Author contributions

NC revised the manuscript. J-YM and HZ collected the data and drafted the manuscript. All authors read the manuscript and approved of the version to be published.

## Abbreviations

AIDS: Acquired Immune Deficiency Syndrome
COVID-19: Corona Virus Disease 2019
FiO_2_: fraction of inspiration O_2_
CT: computed tomography
CTA: computed tomography angiography
FEU: Forty-foot Equivalent Unit
MCA: middle cerebral artery
CTPA: computed tomography pulmonary angiography
cTnI: cardiac troponin I
NT-proBNP: N-terminal pro brain natriuretic peptide
EKG: electrocardiogram
LV: left ventricular
BAL: bronchoalveolar lavage
CRP: C-reactive protein
DVT: deep venous thrombosis.
